# The plant leaf movement analyzer (PALMA): a simple tool for the analysis of periodic cotyledon and leaf movement in *Arabidopsis thaliana*

**DOI:** 10.1186/s13007-016-0153-3

**Published:** 2017-01-03

**Authors:** Lucas Wagner, Christoph Schmal, Dorothee Staiger, Selahattin Danisman

**Affiliations:** 1 Molecular Cell Physiology, Faculty of Biology, Bielefeld University, Bielefeld, Germany; 2 Institute for Theoretical Biology, Charité Universitätsmedizin, Berlin, Germany

**Keywords:** Circadian clock, Iron homeostasis, Rhythmic leaf movement analysis, Rhythmic cotyledon movement analysis, PALMA

## Abstract

**Background:**

The analysis of circadian leaf movement rhythms is a simple yet effective method to study effects of treatments or gene mutations on the circadian clock of plants. Currently, leaf movements are analysed using time lapse photography and subsequent bioinformatics analyses of leaf movements. Programs that are used for this purpose either are able to perform one function (i.e. leaf tip detection or rhythm analysis) or their function is limited to specific computational environments. We developed a leaf movement analysis tool—PALMA—that works in command line and combines image extraction with rhythm analysis using Fast Fourier transformation and non-linear least squares fitting.

**Results:**

We validated PALMA in both simulated time series and in experiments using the known short period mutant *sensitivity to red light reduced 1* (*srr1*-*1*). We compared PALMA with two established leaf movement analysis tools and found it to perform equally well. Finally, we tested the effect of reduced iron conditions on the leaf movement rhythms of wild type plants. Here, we found that PALMA successfully detected period lengthening under reduced iron conditions.

**Conclusions:**

PALMA correctly estimated the period of both simulated and real-life leaf movement experiments. As a platform-independent console-program that unites both functions needed for the analysis of circadian leaf movements it is a valid alternative to existing leaf movement analysis tools.

**Electronic supplementary material:**

The online version of this article (doi:10.1186/s13007-016-0153-3) contains supplementary material, which is available to authorized users.

## Background

Organisms across all kingdoms adapt to the periodic changes of day and night in their environment caused by the rotation of the Earth. Molecular clocks evolved to anticipate dusk and dawn and to synchronize biological processes with the time of the day [1]. In the model species *Arabidopsis thaliana*, plants that are *in sync* achieve higher fitness than plants that are out of phase with the ambient day/night cycle [2].

The Arabidopsis circadian clock is composed of a series of interconnected feedback loops with two related Myb transcription factors, LATE ELONGATED HYPOCOTYL (LHY) and CIRCADIAN CLOCK ASSOCIATED1 (CCA1) and the pseudo response regulator TIMING OF CAB EXPRESSION 1 (TOC1), at its core [3, 4]. The interconnected feedback loops have two functions: they maintain the rhythmic expression of the core clock genes and control the expression of target genes that are involved in a plethora of biological processes [1]. For a comprehensive overview over the composition of the plant circadian clock, see [5, 6].

The endogenous clock of plants is subject to entrainment by inputs that confer information about the external world, i.e. whether it is day or night. Light and temperature are the most commonly known input signals to the plant circadian clock. Another input is photosynthetically supplied sucrose. Addition of external sucrose shortens the period *CCA1* transcript oscillations by 2.7 h [7]. Similarly, iron availability controls the periods of clock genes in a dose-dependent manner [8, 9]. Also, mutants of the FERRIC INDUCED TRANSCRIPTION FACTOR, which are defective in iron homeostasis, exhibit a lengthened period of *CCA1* transcript oscillations [8]. The effect of iron on the circadian clock is dependent on the presence of functional chloroplasts, as plants treated with plastid translation inhibitors, e.g. kanamycine, exhibited no iron-dependent period change of clock gene reporters [8].

The plant clock controls many key biological processes, such as photosynthesis, defence against pathogens, growth, iron homeostasis and leaf movements [9–13]. Periodic leaf movements are among the first described time of day dependent physiological processes in plants. Androsthenes of Thasos, scribe and admiral of Alexander the Great, described ‘sleeping behavior’ of *Tamarindus indica* plants growing on the island of Bahrain [14]. In 1729, the astronomer Jean-Jacques d’Ortous de Mairan demonstrated diurnal rhythms of leaf opening and closing in *Mimosa pudica* plants in day–night cycles that persisted under constant conditions [15]. The first to perform a systematic computer-based analysis of leaf movement rhythms using time-lapse photography in *A. thaliana* were Engelmann et al. [16].

Until today leaf movement experiments are a simple yet powerful way to analyze mutant plants for a circadian clock phenotype. These experiments allow a first assessment whether a specific mutant genotype leads to a deviant clock period in free-running conditions. A frequently used method to estimate leaf movement periods has been developed by Martin Straume and relies on Fast Fourier transformation (FFT) and subsequent non-linear least squares fitting (NLLS) [17]. Based on the FFT–NLLS method, the Millar lab developed the Excel-based Biological Rhythms Analysis Software System (BRASS) [18, 19].

Here, we introduce a simple to use leaf movement analysis tool that combines the detection of leaf movements with the estimation of their periods. The plant leaf movement analyzer (PALMA) functions in command line and requires minimal input from the user. PALMA includes two programs: PALMA1 determines leaf tip positions of plants grown in vertical plates with 25 chambers and PALMA2 calculates the period of the leaf movement rhythms. We tested PALMA in both simulated time series and in wet lab experiments. In the first experiment, we compared cotyledon movements of the known short period mutant, *sensitivity to red light reduced 1* (*srr1*-*1*) with wild type plants [20, 21]. In the second, we tested whether exogenous iron affects periodic leaf movements. Finally, we compared PALMA with BRASS and a newer plant leaf movement analyzer, Tracking Rhythms in Plants (TRiP) [22].

## Results and discussion

Leaf movement rhythms are a commonly known output of the circadian clock [15]. Here, we introduce PALMA, a simple leaf movement analyzer that both extracts leaf movement data from high resolution time-lapse photography and analyses the extracted data using FFT–NLLS.

### PALMA is an easy to use platform-independent console program

PALMA was written in C# using the MonoDevelop framework and hence requires no additional software to perform. It was mainly programmed to work in a Windows environment, although compilation for Linux-systems is possible by using the MonoDevelop compiler. PALMA allows for incorporation into a more automated pipeline because of three reasons: it is designed as a console program, includes an automated leaf tip detection algorithm already, and it is independent from third party software. A small change in the source code can be made to make PALMA respond to console arguments only, removing the necessity for human input.

Updating to different Windows version is obsolete because PALMA works in command line. PALMA has two components: PALMA1 recognizes leaf tips and extracts leaf tip positions for each time point of the time lapse photography. PALMA2 calculates the periods of the leaf movement rhythms using FFT–NLLS.

### PALMA1 identifies the positions of both cotyledon and leaf tips of Arabidopsis seedlings

**Fig. 1:**
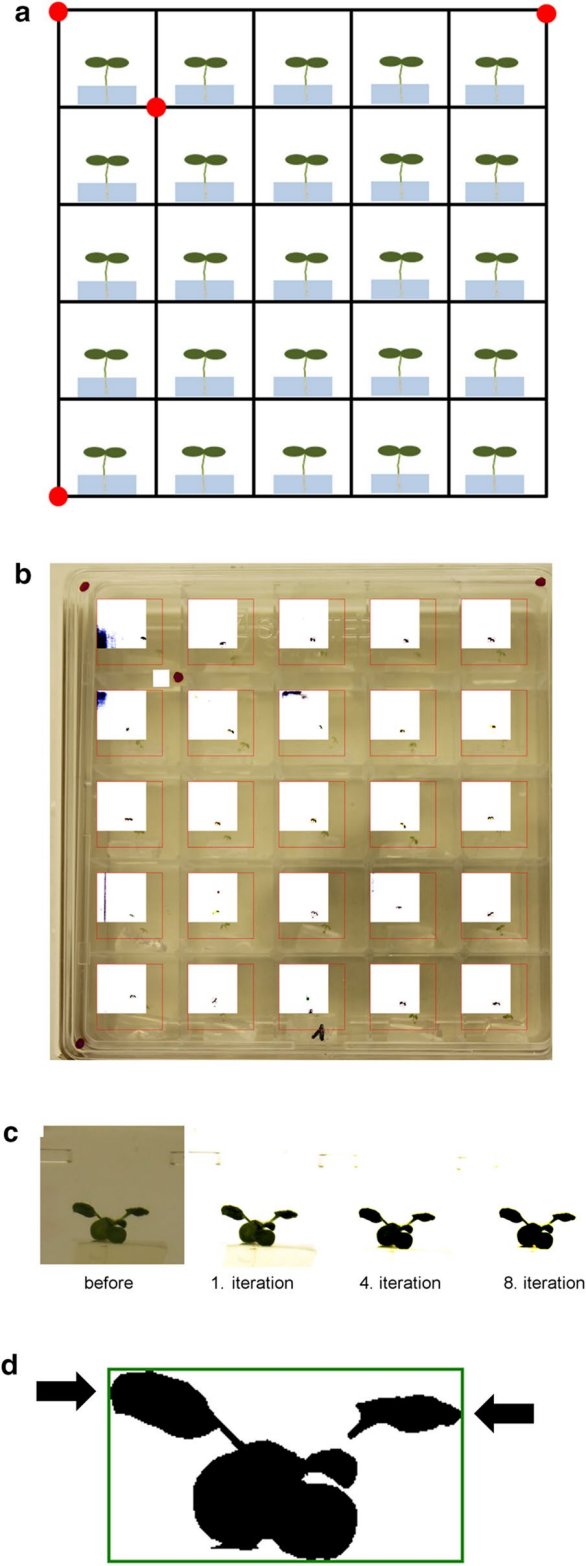
**a** Set-up of leaf movement experiments for recognition by PALMA. 25 seedlings can be analyzed in parallel per vertical plate and each camera takes picture series of one plate. *Red dots* on the plates mark the boundaries of an individual chamber and of the entire plate. **b** Depicts a sample picture of the manual control step allowed by PALMA1. PALMA1 presents and overlay of the recognized chambers (in *white*) with the picture chosen for initial plant detection. *Red squares* indicate the borders of the detected chambers and of detected seedlings. In this example, PALMA1 recognized all chambers correctly but not all seedlings (e.g. seedling in the top left corner). **c** Plant detection by iterative contrasting. The first picture depicts a seedling as seen by camera before contrasting by PALMA1. Pictures two to four depict the same seedling in the first, fourth and eighth iterative contrasting step. **d** Depicts the final plant picture outlined and the detection of leaf tips by those pixels of the binarized seedling picture that cut across the outline (here marked by *arrows*)

PALMA1 recognizes leaf or cotyledon tip positions in time series analysis. When using compartmentalized square dish plates, PALMA1 is able to determine leaf and cotyledon tip positions in each compartment. For the experiments in this study, we used Sterilin™ 100 mm square dishes with 25 compartments. The partition walls between the compartments are transparent. To facilitate compartment detection, PALMA1 was facilitated by marking the dishes with a pattern of red dots. With the help of these red dots, PALMA1 determines both individual chambers and the dimensions of the entire square dish (Fig. 1a; Additional file 1). After this PALMA1 allows for a manual control step in which the correct identification of the individual chambers and the seedling detection can be reviewed (see “Methods” section, Additional file 1) (Fig. 1b). Subsequently, PALMA1 recognizes the individual plants by the contrast between the plant and the background. We avoided a detection algorithm based on green coloration to allow for plants with different chlorophyll contents (Fig. 1c). This contrasting is performed in eight iterations and leads to a black-and-white picture that is used for subsequent leaf tip detection. PALMA1 detects leaf tip positions of every picture in a time lapse series by tracing the left and right rims of each plant, respectively (Fig. 1d). PALMA1 can identify both leaf tips and cotyledon tips using this simple algorithm provided that the individual leaves/cotyledons are placed exactly orthogonal to the viewing direction of the camera. Such a careful placement of the seedlings increases the success rate of leaf and cotyledon tip detection by PALMA1.

### PALMA2 correctly determines periods and phase shifts in simulated time series

**Fig. 2:**
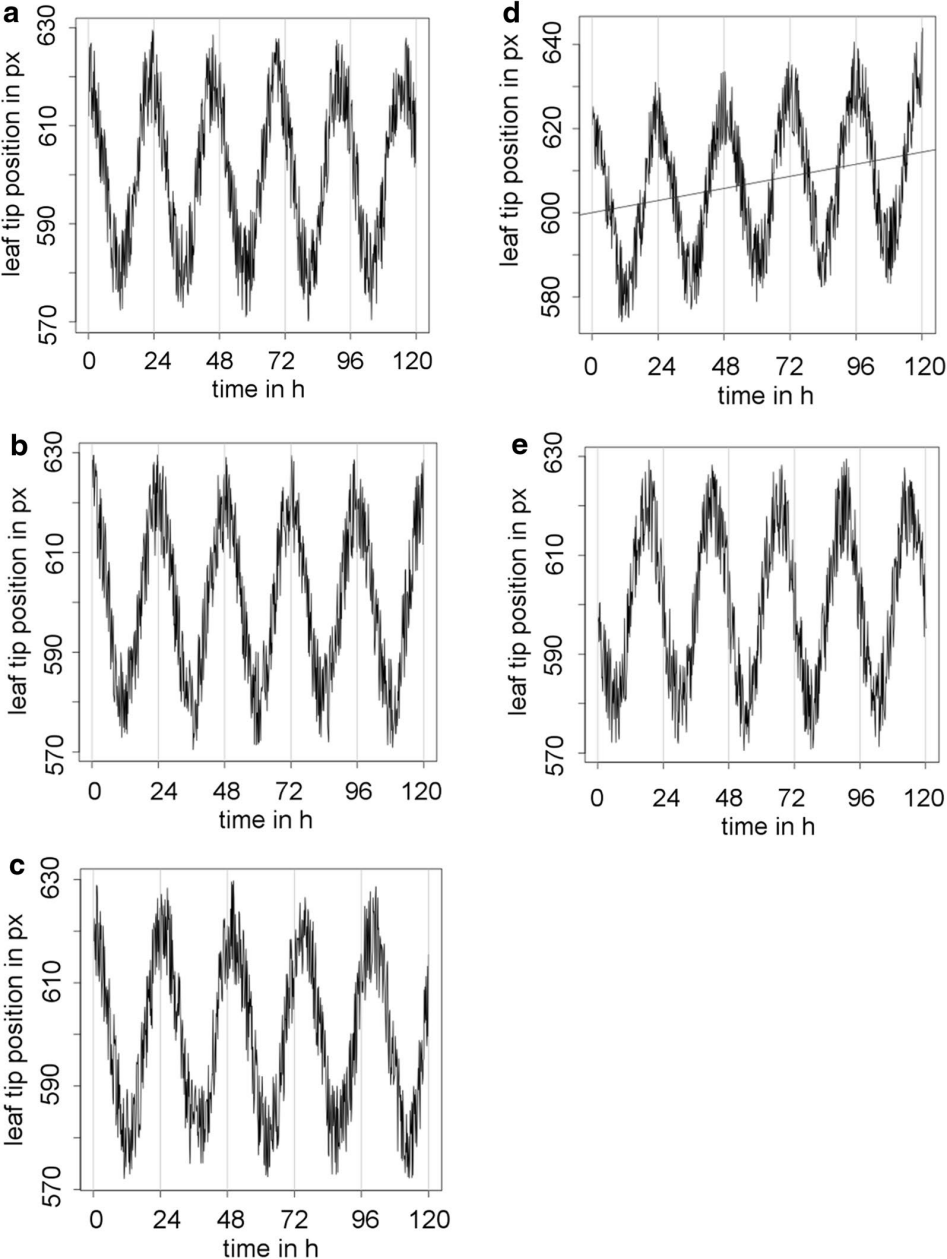
Simulated time series for leaf movement analyses. **a**–**c** Time series with a period of 23, 24, and 25 h, respectively. **d** Time series with a linear growth trend (*blue line*). **e** Time series with a 6 h phase shift

After identification of leaf or cotyledon tip positions by PALMA1, PALMA2 estimates periods and phases of the leaf movements. We first tested whether PALMA2 correctly determines periods and phases in simulated leaf movement rhythms. Time series simulations were created by generating a simple cosine wave-function using average leaf movements. Noise (±15%) was added to the time series to simulate biological variance (Fig. 2a–e). We tested PALMA2 using five time series. Three time series represented leaf movements with circadian periods of 23, 24, and 25 h, respectively. PALMA2 correctly predicted the periods of these time series as 23.26, 23.99 and 25.05 h, respectively. The fourth time series represented a 24 h-rhythm with a linear growth trend simulating plant growth over the course of the experiment (Fig. 2d). Also here, PALMA2 correctly identified the period as 23.99 h and hence corrected for the linear growth trend. The fifth time series represented leaf movements of a 24 h period but with a 6 h phase delay in comparison to the simple wave function used initially (Fig. 2e). Here, PALMA2 identified the correct period despite the 6 h phase shift (Table 1).

**Table 1:** Performance of PALMA2 in the analysis of simulated leaf movement time series in comparison to BRASS and TRiP

Data set	Simulated phenotype	PALMA	BRASS	TRiP
1	23 h period	23.26 h	22.97 h	22.99 h
2	24 h period	23.99 h	24.00 h	24.01 h
3	25 h period	25.05 h	24.99 h	25.01 h
4	24 h + linear growth trend	23.99 h	24.00 h	24.05 h
5	24 h + 6 h	24.00 h + 6 h	23.62 h + 6 h	24.06 h + 6.3 h

**Fig. 3:**
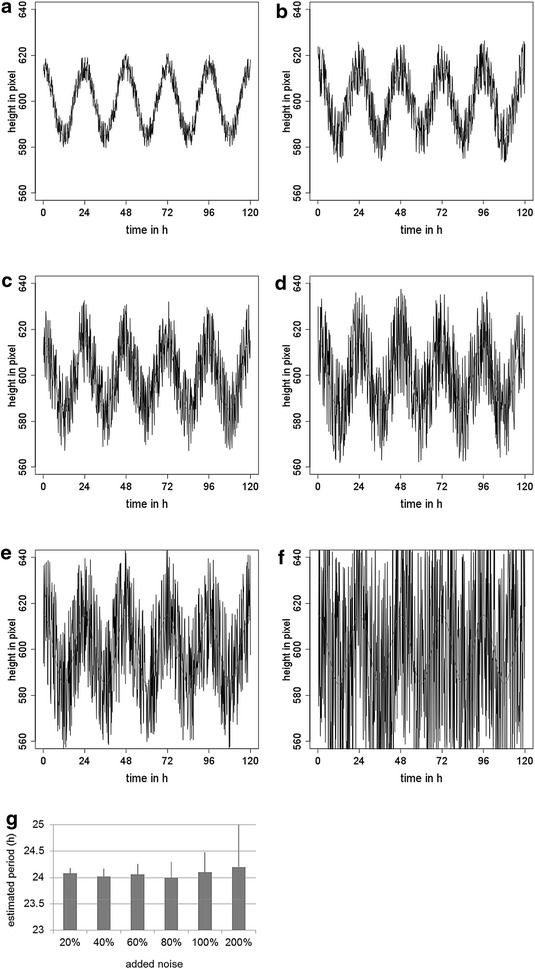
Effect of noise on PALMA2 performance. **a**–**f** Outputs of PALMA2 when tested with simulated leaf movement data with 20, 40, 60, 80, 100 and 200% added noise, respectively. *Black lines* represent simulated leaf movement data, *red lines* the calculated period. Histogram (**g**) summarizes the results of (**a**–**f**), showing calculated periods and standard deviations for each noise condition tested

In another series of tests we analysed the sensitivity of PALMA2 period estimation to increased noise. We simulated the noisiness of real leaf rhythm analyses by adding evenly distributed noise to a simulated leaf movement rhythm of 24 h. The tested noise intensities were 20, 40, 60, 80, 100 and 200% noise, respectively (Fig. 3a–f). In four out of five cases, PALMA2 recognized the 24 h period correctly. At a noise of 200%, the standard deviation was too high and a correct period could not be estimated by PALMA2 (Fig. 3g).

### Comparison with established leaf movement analysis programs

We subsequently compared the performance of PALMA2 with the performances of two other leaf movement programs, BRASS and TRiP [18, 22]. BRASS performs only in conjunction with the proprietary software Microsoft Excel and thus only operates on Windows systems. In addition, BRASS itself does not extract leaf movement data from time lapse pictures and thus needs to be combined with other programs [18]. The Millar lab generally uses the commercially available image analysis software MetaMorph™. Even using different image analysis software, conversion between generated data and BRASS-feasible input data is complex and requires additional processing steps. Incorporating BRASS in a computational pipeline is difficult, as the original BRASS program does not allow for batch processing and requires additional scripting efforts.

TRiP relies on the proprietary MATLAB computing environment or its UNIX equivalent Octave [22]. While its dependence on MATLAB makes it somewhat inflexible, the use of MATLAB allows for easy customisation of the given program. Although TRiP can be incorporated into a pipeline, PALMA’s automated plant position acquisition algorithm allows for an easier incorporation into pipelines. Overall, both TRiP and PALMA are valid alternatives to BRASS. As TRiP uses dynamic image acquisition, it can potentially determine leaf movement rhythms better than PALMA with its simple leaf tip detection algorithm. However, the necessity of the manual assessment of plant positions makes it impracticable for use in large data scaling [22]. PALMA’s command line nature allows for easy customization into new platforms and can be used immediately on computers without the need for installation or licencing issues.

We performed rhythm analyses of the same time series simulations with BRASS and TRiP and found that they performed similarly to PALMA. In all cases, short period and long period mutants were identified correctly. Also all programs performed similarly well on data containing a linear growth trend and a phase delay of 6 h. The simulated time series experiment shows that PALMA2 conducts correct FFT and NLLS and can be used to determine periods of leaf movement experiments.

### PALMA correctly identified a short period mutant in wet-lab experiments

**Fig. 4:**
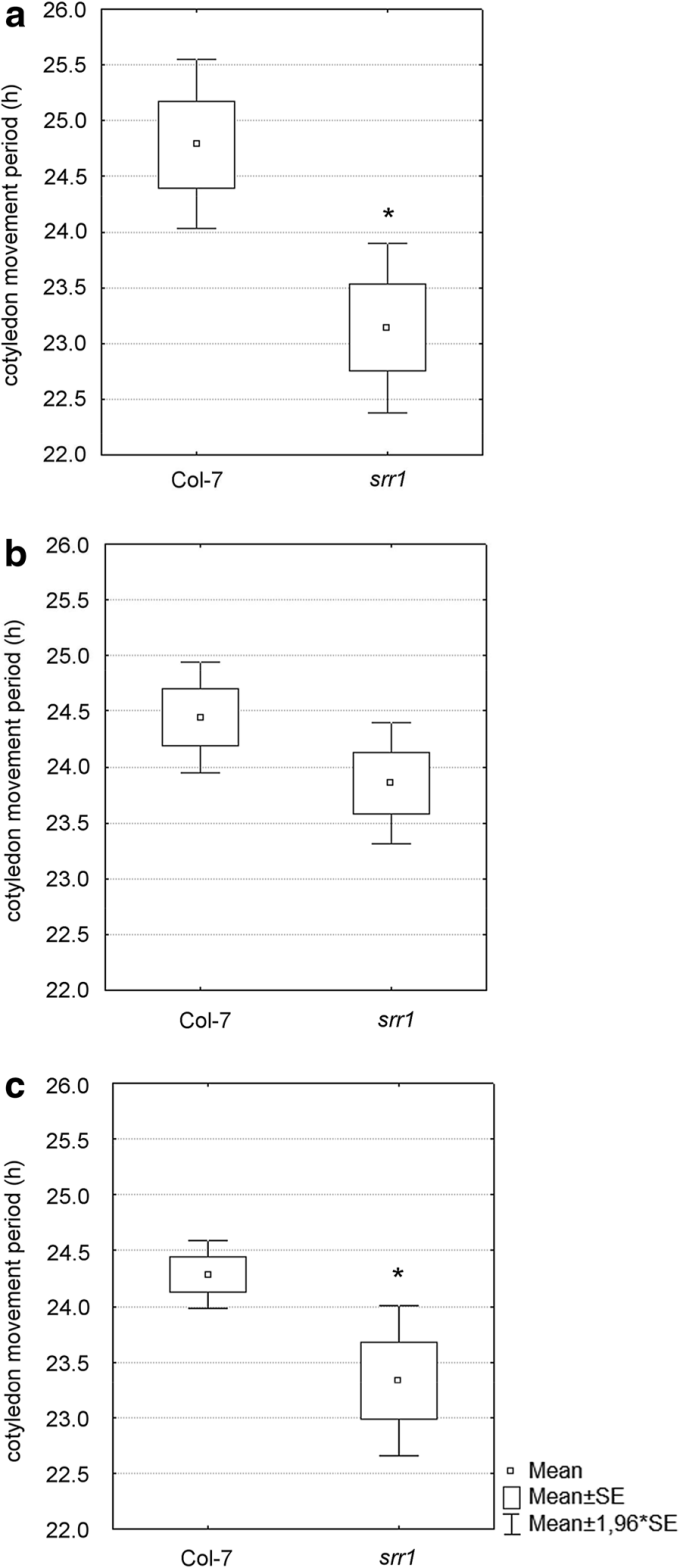
Leaf movement rhythms of the *srr1*-*1* mutant and the corresponding Col-7 wild type analyzed by **a** PALMA, **b** BRASS [18], and **c** TRiP [22] (n = 40). *Asterisks* indicate statistical significance (p < 0.05) in a Student’s t test

Having shown that PALMA2 correctly identifies phases and periods of simulated time series, we tested PALMA under laboratory conditions. For this we used the short period mutant *srr1*-*1*. SRR1 is involved in phytochrome B-mediated signalling and the mutant exhibits a shorter period of leaf movements as well as shorter periods of core clock gene expression, such as *CCA1*, *LHY* and *TOC1* as well as of output gene expression [20, 21]. Seedlings of *srr1*-*1* and the corresponding Col-7 wild type were grown for four days in a 12/12 day–night regime after which they were transferred into the vertical chambers and into continuous light conditions for time lapse photography. Whereas the Col-7 wild type plants showed a cotyledon movement period of 24.7 h, the *srr1*-*1* mutant displayed significantly reduced cotyledon movement periods of 23.14 h (p = 0.005) (Fig. 4a). This period shortening is similar to earlier observations using a Kujata imaging system and subsequent FFT–NLLS analysis [20]. We next analysed the same experiment using BRASS and TRiP. BRASS does not include a leaf detection program, so the leaf tip positions determined by PALMA1 were used for period estimation by BRASS. TRiP does include leaf detection and used the leaf positions determined by its own algorithm. Further, TRiP uses a Nelder–Mead nonlinear optimization algorithm to fit the single FFT component [23]. All programs detected the same reduction in cotyledon movement periods although BRASS could not find a significant effect in the data (p = 0.13) (Fig. 4b). As BRASS used the same leaf tip positions as PALMA2, the difference in the significance is mainly due to the detrending algorithm that differs between BRASS and PALMA2. While BRASS uses a two-pass detrending using a weighted gaussian kernel, PALMA2 uses a simple one-pass moving average algorithm. In the Col-7 data set, TRiP determines a standard deviation of 0.58 h (Fig. 4c) whereas both BRASS (1.02 h) and the PALMA (1.6 h) analyses lead to higher standard deviations. Thus, PALMA performs similarly to both TRiP and BRASS.

All three programs use common time lapse photography to analyse leaf movements. Recently, Dornbusch et al. [24] developed a three-dimensional system which was able to discern the effects of leaf growth and leaf blade position on the leaf movements of Arabidopsis. These detection methods are superior to simple time lapse photography because they follow leaf movements in all three dimensions, allowing for the analysis of atypical leaf movements. PALMA was designed for simple detection of circadian clock mutants in Arabidopsis and although it can detect leaf movements both on the horizontal and the vertical axis, we limited the PALMA1 output to movements on the vertical axis, only.

### Reduced iron availability leads to shortened leaf movement periods in Arabidopsis

Low iron leads to longer periods of circadian clock gene expression in Arabidopsis [8, 25]. We used PALMA to investigate whether this period lengthening under reduced iron conditions is detectable also for leaf movement rhythms. For this, wild type plants were grown in a 12 h/12 h day/night rhythm on medium containing 100 µM Fe(III)-EDTA for one week. After this, the seedlings were transferred to medium containing 100 µM Fe(III)-EDTA (i.e. iron-rich conditions) or 20 µM Fe(III)-EDTA (i.e. iron-limited conditions), respectively. After transfer the seedlings were entrained for one more day to allow adjustment to the new medium. The next day the seedlings were transferred into the vertical leaf movement analysis chambers and into continuous light conditions for time lapse photography. In contrast to the *srr1* experiment, the seedlings were positioned to allow identification of the leaf tip positions of the first leaves instead of cotyledon tips. As the seedlings were transferred after seven days instead of four days, the growing first leaves would have impeded periodic cotyledon movements. Hence it was easier to detect period movements in the first leaves than in the cotyledons. We also noticed that plants that grew on 20 µM Fe(III)-EDTA were more likely to exhibit difficult to analyse leaf movement behaviour, as also the growth of the plants was impeded after transfer from 100 to 20 µM Fe(III)-EDTA. About 50% of all seedlings that were transferred had to be discarded before the statistical analysis (n = 32).

**Fig. 5:**
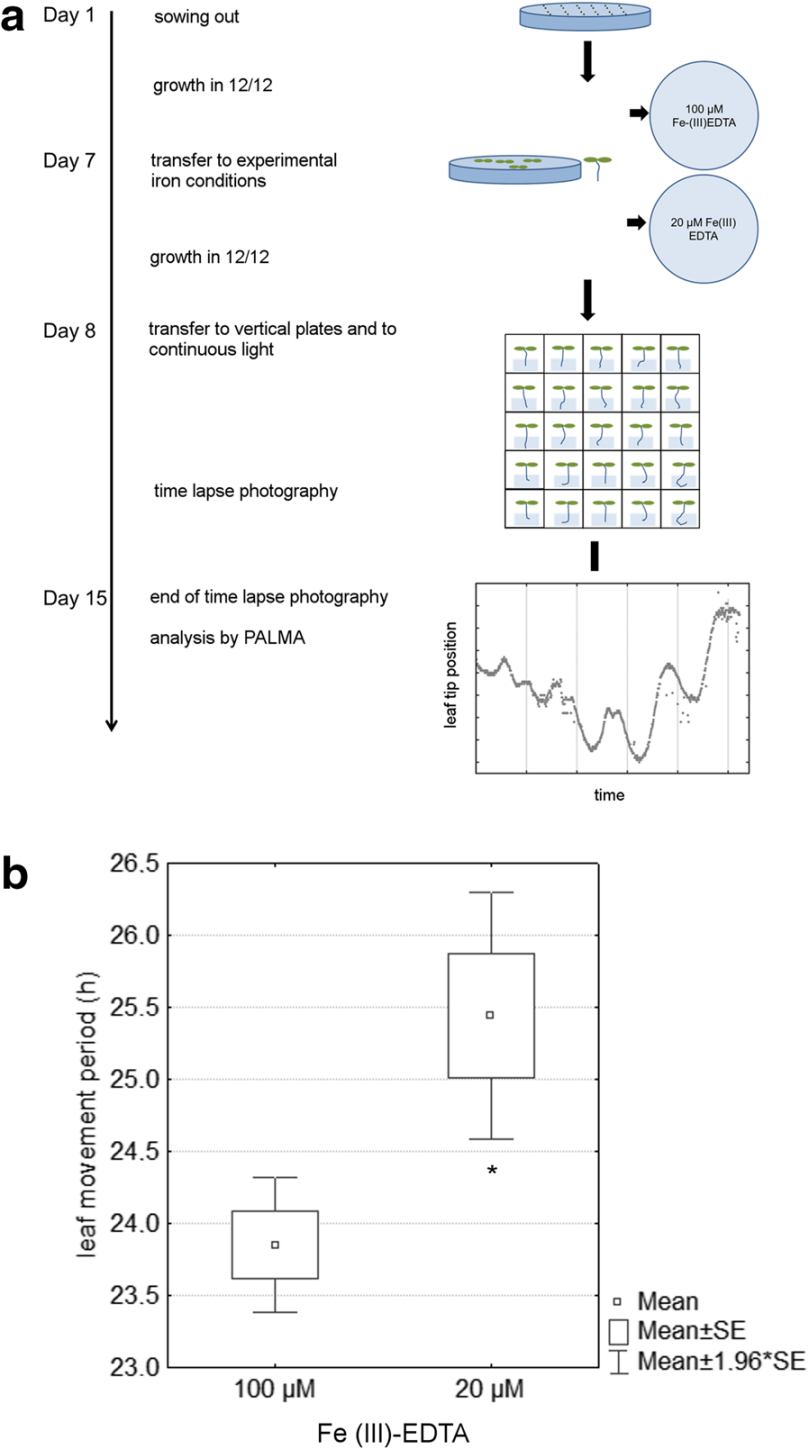
Analysis of iron effects on Arabidopsis leaf movement rhythms. **a** Experimental set-up of the experiment. **b** Period of leaf movements of Col-0 wild type plants on agar containing 100 µM and 20 µM Fe(III)-EDTA, respectively. The *asterisk* marks a significant difference in a Student’s t test [p = 0.0007; n = 62 (100 µM), n = 31 (20 µM)]. The experiment was repeated twice with similar results

We found that seedlings that were transferred 20 µM Fe(III)-EDTA showed a significant increase of 1.59 h in leaf movement periods (Student’s *t* test; p = 0.0007) (Fig. 5). Thus, iron also affects leaf movement rhythms, and reduced iron lengthens the period, as previously observed for clock gene expression. This is in accordance with the expected effect of reduced iron on the circadian clock, where reduced iron led to a period of clock gene expression that was lengthened between 30 min on minimum medium without iron and 2 h on medium containing Ferrozine as iron chelator [25]. In another study, the period of TOC1:luciferase oscillations lengthened by almost 3 h between 100 and 0.25 µM iron concentration in the medium [8]. This means that iron also affects periodic leaf movements and that reduced iron lengthens the period of these.

Overall, leaf movement rhythms are another output of the clock that is dependent on iron. The effect of iron on the clock can be studied by time lapse photography and subsequent rhythm analysis by PALMA, in addition to monitoring clock gene expression using e.g. a promoter-luciferase fusion construct.

## Conclusions

The new and simple plant leaf movement analysis tool PALMA can estimate the correct period in both simulated and real leaf movement experiments. It performs similarly to established leaf movement analyzers [18, 22], with the advantage of being able to extract leaf movement data from images and analysing these while remaining platform independent (Table 2). Using PALMA we found that reduced iron conditions lengthen the period of leaf movement rhythms in Arabidopsis, an effect of reduced iron availability that is similar to what has been observed on the period of clock gene expression in earlier studies [8, 25].

**Table 2:** Comparison of system requirements, functions and performance between BRASS, PALMA and TRiP

	BRASS	PALMA	TRiP
Plant detection	None	Leaf tip detection	Dynamic
Mathematical analysis	FFT–NLLS	FFT–NLLS	Nelder–Mead optimization of FFT
Environment	Windows Excel	Command line	MATLAB
Performance on in silico data	Similar	Similar	Similar
Performance on *srr1*-*1* data	Similar	Similar	Similar

## Methods

### Plant material

Col-7 and *sensitivity to red light reduced 1* (*srr1*-*1*) were described before [20, 21]. Col-0 was used to test the effect of iron on periodic leaf movements.

### Leaf movement analyses

For testing the performance of PALMA on *srr1*-*1* plants, seeds were sown out on half-strength Murashige–Skoog plates [26] and grown at a day–night rhythm of 12 h/12 h in a PERCIVAL growth chamber for four days. After four days, seedlings were transferred by cutting a roughly 1 × 1 cm square into the agar around the individual seedlings and transferring the seedlings together with the agar blocks into Sterilin™ 100 mm square petri dishes with 25 compartments (Thermo Scientific). Seedlings of the different genotypes were placed into the compartments in a randomized manner. All seedlings were placed in the middle of the respective chamber in a way that both cotyledons were detectable, i.e. orthogonal to camera-angle. The plates were covered with two lids on the side facing the camera to avoid condense water accumulation in form of droplets. The plates were immediately placed into continuous light and pictures were taken from the first day onwards.

For testing the effect of iron on leaf movement periods, Col-0 seeds were grown on self-made medium consisting of 2 mM Ca(NO_3_)_2_, 0.75 mM K_2_SO_4_, 0.65 mM MgSO_4_, 0.1 mM KH_2_PO_4_, 10 µM H_3_BO_3_, 0.1 µM MnSO_4_, 0.05 µM CuSO_4_, 0.05 µM ZnSO_4_, 0.005 µM (NH_3_)_6_Mo_7_O_24_, 0.5 g MES, 100 µM Fe(III)-EDTA, 0.5% sucrose, pH 5.7and 1% agar. The seedlings were grown in a 12/12 day/night rhythm for seven days before transfer of the seedlings to 20 µM Fe(III)-EDTA (low iron). As control, half of the seedlings were transferred to 100 µM Fe(III)-EDTA. After transfer the seedlings were grown one additional day at a 12/12 rhythm before being transferred into the leaf movement experiment. For the leaf movement analysis, the first leaves of mutant and wild type plants were photographed.

### Camera settings

For all leaf movement experiments, Canon EOS 1100D high resolution cameras were used. Cameras and plates were fixed to avoid positional changes in the course of the experiment. White paper was affixed behind the plates to increase the contrast of the plants to the background. Three cameras were controlled by one computer using the Linux-based program *gphoto2*. Focus settings were set on manual and checked before each experiment started. Pictures were taken for seven days at a rate of one picture every 10 min.

### PALMA1: determination of cotyledon and leaf tip positions

The underlying framework for seedling recognition was ‘AForge.NET Framework’ [27] that was developed for research in picture recognition for artificial intelligences. The framework for Fourier analyses and multiplication of vectors and matrices is the ‘Math.NET’ framework (Additional file 2). PALMA extracts the position of the leaf tips from the images of the time lapse photography using a threshold binarization algorithm, coupled with a simple blob detection algorithm. The experimental setting was marked using red dots to help PALMA identify plant positions and tilting angle of the plates (Fig. 1). The red dots indicate the number of compartments in which plants need to be identified by PALMA1. Whereas we used Sterilin™ 100 mm square petri dishes with 25 compartments (Thermo Scientific), placement of the red dots is flexible and thus able to detect a variable number of compartments (see Additional file 1). For this, the plates are marked using four red dots at specific positions that define both the single chambers and the whole plate (Fig. 1a). PALMA1 recognizes the red marks using a radial hue, saturation and value (HSV)-colour filter. The filter works by projecting the presented colours in the 360° HSV colour space and selecting the pixels that are red (hue value: 0°). PALMA1 allows for 15° deviations from 0° to tolerate different hues of red. After filtering for red marks, PALMA1 binarizes the pictures and subsequently removes noise by erosion and dilation. Then PALMA1 uses a blob detector with a size filter that excludes all signals smaller than 0.5% of the overall picture’s size. Finally, PALMA1 determines the positions of all chambers using the four red marks. As the red marks are placed manually and camera settings, camera position and the presence of noise can still lead to incorrect detection of chambers, PALMA1 allows for a manual control step. In case that PALMA1 did not recognize all chambers correctly, users can correct the mark positions on one sample picture using a simple graphic tool and feed PALMA with this new information.

Once the chambers were detected successfully, PALMA1 determines the outlines of all seedlings in these chambers. For this, PALMA1 reduces the picture size of all seedling chambers by 30% to further reduce noise. Plants are detected based on their contrast to the background and not their green colour to allow analysis of plants with different chlorophyll contents. Plant detection by contrast is made possible by luminescence filtering in eight iterations (Fig. 1b). First all images are adjusted to an even average luminescence level to allow the luminescence filtering to perform evenly on each image. Then the colour spectrum is sheared linearly by using the average luminescence level of the image as a threshold in eight iterations. As the major part of the image is expected to be background colour, the average luminescence level is expected to group slightly below the majority of background luminescence. The difference between foreground and background is increased by linearly mapping the lower luminescence spectrum (zero to mean) to the whole spectrum. A contrast correction of 10% is used to increase the difference in each step. After eight iterations the image is binarized into a black-and-white picture and used for image detection.

For each binarized image simple blob detection is used to locate an initial plant position. As the luminescence filtering can cause interference factors (like speckles, water droplets, etc.) to surface more prominently and plants can appear segmented after filtering, the located plant blobs are revised to accurately locate the plants. As this can lead to individual leaves being separate blobs, all blobs found in the image are united using a metric filter which identifies plant structures based on the blob’s distance to the image centre and the size and the form of the blob.

After locating each plant, PALMA1 detects the leaf tip movements by tracing the left and right rim of each plant (Fig. 1c). By averaging all data points found within 3–5 pixels vicinity to the borders the left and right leaf tip position can be reasonably approximated. In Arabidopsis, both cotyledon movements and movement of the first leaves can be detected using this method and depending on the preferences of the individual experiment. Whereas time lapse photography allows for a detection of leaf tips in both x- and y-axis, period estimation by PALMA2 only includes movements of the y-axis. Hence, PALMA1 only determines the movements of leaf tips in the y-axis. For this, camera set-ups need to capture leaf positions in three dimensions. Below, we performed both analyses of cotyledon movements and of first leaves. Whereas cotyledons allow for a faster analysis as time lapse photography can start at day four after germination, some experimental set-ups make the analysis of first leaves more reasonable. The analysis of the movement rhythms of first leaves allows using older seedlings in which cotyledon movements are largely dampened once the first leaves emerge. Finally, the positions of all left and right leaf tips are stored in a.csv-file and then used in the rhythm analysis by PALMA2.

### PALMA2: determination of periods

Since the movements of the leaf tips follow circadian rhythms, the fitting function is assumed to be a summation of multiple cosine terms, with each term denoting one prominent set of amplitude, period and phase [17]. By iteratively applying the Gauss–Newton method an accurate approximation of the data was generated from an inaccurate guess for the given fitting function. In rhythmic functions with a time-independent period, the experimental data can be approximated to a high precision by using a summation of multiple cosine terms. We found that expansion of the fitting function to less than 5 terms often suffices for a good approximation. PALMA2 stops the fitting when the variance of next added term is above 0.1. Then PALMA2 removes the linear growth trend of the plants using simple linear regression. A FFT is run on the data to receive an initial estimation of the fitting parameters which is further optimized by NLLS fitting using the Gauss–Newton method. To define the precision of the parameters, the approximated nonlinear support plane joint confidence interval is calculated for each parameter [17]. Using this approximated confidence interval, relative errors of each parameter can be calculated. Using this error for each set of parameters (of amplitude, period and phase) the period of leaf movements can be identified. The period with the least relative error and hence the cosine term which has the most impact on the fitting was consequently determined as the leaf movement period of the individual plant [17, 18]. Due to the decentralized nature of the plant circadian clock [28], leaf rhythms can vary considerably between the left and the right leaves of the same plant. Whereas TRiP solves this problem by averaging all determined periods [22], we do not recommend averaging the right and left leaf tip periods determined by PALMA2. This is mainly due to the fact that in several cases only one of the two leaf tips generates a reliable period. Averaging a reliably determined period with a noisy period will generally lead to a more erroneous estimate. Hence, we selected always the left leaf of an individual plant’s time lapse photos and only selected the right leaf when there was no period to be determined from the left leaf.

## Additional files

**Additional file 1.** Walkthrough.

**Additional file 2.** Programme files.

## Abbreviations

BRASS: Biological Rhythm Analysis Software
CCA1: CIRCADIAN CLOCK ASSOCIATED 1
FFT: Fast Fourier transformation
LHY: LATE ELONGATED HYPOCOTYL
NLLS: non-linear least squares fitting
PALMA: plant leaf movement analyzer
SRR1: SENSITIVITY TO RED LIGHT REDUCED 1
TOC1: TIMING OF CAB EXPRESSION 1
TRiP: Tracking Rhythms in Plants

## References

[1] Greenham K, McClung CR (2015). Integrating circadian dynamics with physiological processes in plants. Nat Rev Genet.

[2] Green RM, Tingay S, Wang Z-Y, Tobin EM (2002). Circadian rhythms confer a higher level of fitness to Arabidopsis plants. Plant Physiol.

[3] Schaffer R, Ramsay N, Samach A, Corden S, Putterill J, Carré IA (1998). The late elongated hypocotyl mutation of Arabidopsis disrupts circadian rhythms and the photoperiodic control of flowering. Cell.

[4] Wang ZY, Kenigsbuch D, Sun L, Harel E, Ong MS, Tobin EM (1997). A Myb-related transcription factor is involved in the phytochrome regulation of an Arabidopsis Lhcb gene. Plant Cell.

[5] Hsu PY, Harmer SL (2014). Wheels within wheels: the plant circadian system. Trends Plant Sci.

[6] Staiger D, Shin J, Johansson M, Davis SJ (2013). The circadian clock goes genomic. Genome Biol.

[7] Haydon MJ, Mielczarek O, Robertson FC, Hubbard KE, Webb AAR (2013). Photosynthetic entrainment of the *Arabidopsis thaliana* circadian clock. Nature.

[8] Salomé PA, Oliva M, Weigel D, Krämer U (2012). Circadian clock adjustment to plant iron status depends on chloroplast and phytochrome function. EMBO J.

[9] Zhang C, Xie Q, Anderson RG, Ng G, Seitz NC, Peterson T (2013). Crosstalk between the circadian clock and innate immunity in Arabidopsis. PLoS Pathog.

[10] Cumming BG, Wagner E (1968). Rhythmic processes in plants. Annu Rev Plant Physiol.

[11] Korneli C, Danisman S, Staiger D (2014). Differential control of pre-invasive and post-invasive antibacterial defense by the Arabidopsis circadian clock. Plant Cell Physiol.

[12] Sellaro R, Pacín M, Casal JJ (2012). Diurnal dependence of growth responses to shade in Arabidopsis: role of hormone, clock, and light signaling. Mol Plant.

[13] Duc C, Cellier F, Lobréaux S, Briat J-F, Gaymard F (2009). Regulation of iron homeostasis in *Arabidopsis thaliana* by the clock regulator time for coffee. J Biol Chem.

[14] Bretzl H (1903). Botanische Forschungen des Alexanderzuges.

[15] De Mairan J. Observation Botanique. Hist. L’Academie R. Sci. Paris. 1729;35.

[16] Engelmann W, Simon K, Phen CJ (1992). Leaf movement rhythm in *Arabidopsis thaliana*. Z Für Naturforschung C J Biosci.

[17] Straume M, Frasier-Cadoret S, Johnson M. Least-squares analysis of fluorescence data. In: Lakowicz JR, editor. Topics in fluorescence spectroscopy. New York: Springer; 1991. p. 177–240.

[18] Locke JCW, Southern MM, Kozma-Bognár L, Hibberd V, Brown PE, Turner MS (2005). Extension of a genetic network model by iterative experimentation and mathematical analysis. Mol Syst Biol.

[19] Southern MM, Millar AJ. Circadian genetics in the model higher plant, *Arabidopsis thaliana*. Methods Enzym. Academic Press; 2005 (cited 2015 Nov 11). p. 23–35. http://www.sciencedirect.com/science/article/pii/S0076687905930024. 10.1016/S0076-6879(05)93002-4 15817285

[20] Staiger D, Allenbach L, Salathia N, Fiechter V, Davis SJ, Millar AJ (2003). The Arabidopsis SRR1 gene mediates phyB signaling and is required for normal circadian clock function. Genes Dev.

[21] Johansson M, Staiger D (2014). SRR1 is essential to repress flowering in non-inductive conditions in *Arabidopsis thaliana*. J Exp Bot.

[22] Greenham K, Lou P, Remsen SE, Farid H, McClung CR (2015). TRiP: Tracking Rhythms in Plants, an automated leaf movement analysis program for circadian period estimation. Plant Methods.

[23] Nelder JA, Mead R (1965). A simplex method for function minimization. Comput J.

[24] Dornbusch T, Michaud O, Xenarios I, Fankhauser C (2014). Differentially phased leaf growth and movements in arabidopsis depend on coordinated circadian and light regulation. Plant Cell.

[25] Hong S, Kim SA, Guerinot ML, McClung CR (2013). Reciprocal interaction of the circadian clock with the iron homeostasis network in Arabidopsis. Plant Physiol.

[26] Murashige T, Skoog F (1962). A revised medium for rapid growth and bio assays with tobacco tissue cultures. Physiol Plant.

[27] Kirillov A. AForge. NET framework; 2013.

[28] Endo M, Shimizu H, Nohales MA, Araki T, Kay SA (2014). Tissue-specific clocks in Arabidopsis show asymmetric coupling. Nature.

