# Supplementary material for: The plant leaf movement analyzer (PALMA): a simple tool for the analysis of periodic cotyledon and leaf movement in Arabidopsis thaliana
Source: Plant Methods. 2017 Jan 3;13:2. doi: 10.1186/s13007-016-0153-3 (PMC5209843; doi:10.1186/s13007-016-0153-3)

**Supplemental file:**

**Plant Leaf Movement Analyser – PALMA**

**Walkthrough**

**PALMA1 – Determination of leaf tip positions**

1. Open the PALMA1 program by double-clicking on the Application file
2. PALMA1 asks you whether you want to perform a leaf extraction, if so, type “y” or “yes”
3. Put in the directory of the time lapse photography pictures to be analysed. All photos should be in one directory and there should be no other files in the directory
4. PALMA1 asks you whether you chose the right directory, if so, type “y”
5. PALMA1 will compartmentalize the first picture in the series (or a picture manually given by the user) according to the visible red dots on the picture (Figure 1). The compartmentalization relies on correct placement of the red dots. PALMA1 creates a sample picture that needs to be reviewed by the user.
6.
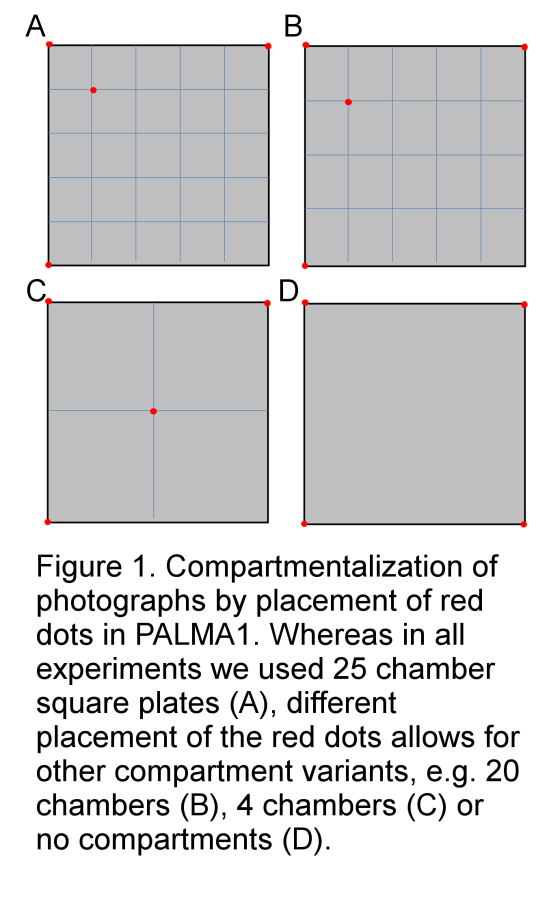
If the compartments are wrongly placed, the user needs to rearrange the red dots on the chosen picture using a simple image manipulation program (Paint, Gimp etc.) and ask PALMA1 to perform the sample analysis again.
7. Once the compartmentalization is sufficient, PALMA1 asks for a folder to place the result files in.
8. Then PALMA1 starts with image excision and determination of the right and left leaf tips of all pictures in the image series.
9. PALMA1 saves the results in the format “1.csv”,”2.csv”, etc. beginning with the upper left compartment, then the one next in the row, until all compartments are analysed. These are the files PALMA2 works with.
10. To prevent PALMA2 from crashing, it is important to review the csv-files for columns of zero’s, an indication that leaf tip detection went wrong in a specific sample. It is recommended to remove these samples from the analysis.

**PALMA2 – calculation of leaf movement periods**

1. Start PALMA2 by double-clicking on the program file.
2. Chose the folder in which PALMA2 will find the csv-files
3. Chose the folder in which PALMA2 places the results files
4. Next, PALMA2 asks for the cut-off, i.e. the number of pictures in the beginning and the end of the time series that will be removed from rhythm analysis. The user can also manually remove pictures, either before PALMA1 analyses the pictures or by deleting the respective rows in the csv-files that are used by PALMA2, but we to use one cut-off for all samples.
5. PALMA2 now performs the rhythm analyses and produces a result file for every leaf tip (two per plant), showing the detected pixel positions of the leaf tip during the time lapse experiment (red), the calculated best fit curve (black) (Figure 2). PALMA2 shows the calculated amplitudes (dimensionless), phases and, most importantly, periods of the leaf movement for every fit it has performed on the data until there was no influence of additional fits to the results. Usually the first fit represents best the overall period of the leaf movements, which is also apparent in a high amplitude and low amplitude error. Users are able to manually assess the best fit by determining the periods in the graphs. In the example the best fit is the first with a determined period of 24.96 hours (Figure 2).


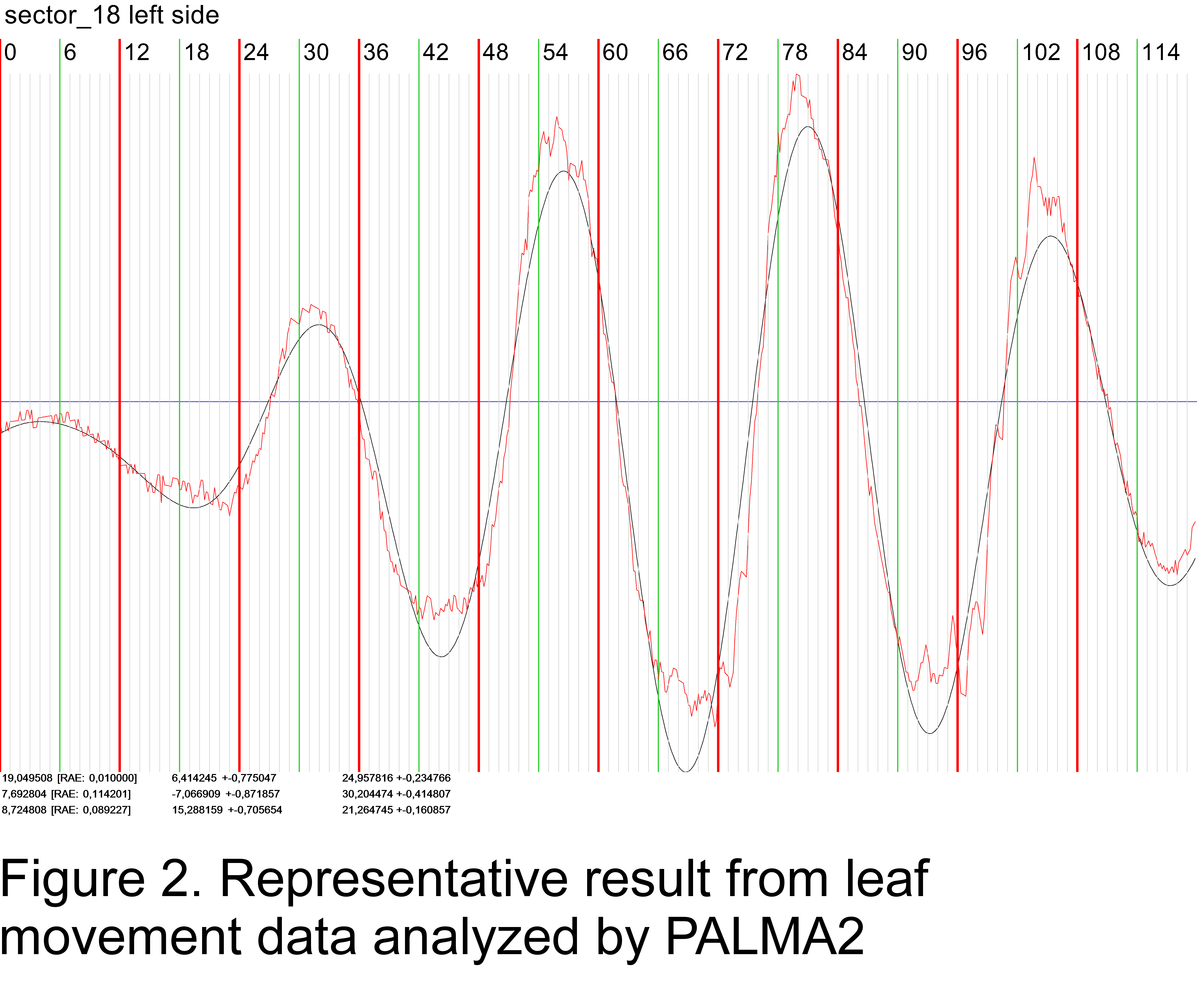

Supplement: Supplementary file 1 — Additional file 1. Walkthrough. [file 13007_2016_153_MOESM1_ESM.docx]
